# BCAP31, a cancer/testis antigen-like protein, can act as a probe for non-small-cell lung cancer metastasis

**DOI:** 10.1038/s41598-020-60905-7

**Published:** 2020-03-04

**Authors:** Jing Wang, Dongbo Jiang, Zichao Li, Shuya Yang, Jiayi Zhou, Guanwen Zhang, Zixin Zhang, Yuanjie Sun, Zhipei Zhang, Xiaofei Li, Liang Tao, Jingqi Shi, Yuchen Lu, Lianhe Zheng, Chaojun Song, Kun Yang

**Affiliations:** 1 Department of Immunology, the Fourth Military Medical University, No.169 Changle W. Rd., Xi’an, 710032 China; 2 Department of Thoracic Surgery, Tangdu Hospital, the Fourth Military Medical University, No.169, Changle W. Rd., Xi’an, 710032 China; 3 Department of Orthopedics, Tangdu Hospital; the Fourth Military Medical University, No.169, Changle W. Rd., Xi’an, 710032 China

**Keywords:** Cancer, Lung cancer, Non-small-cell lung cancer

## Abstract

Non-small-cell lung cancer (NSCLC) represents most of lung cancers, is often diagnosed at an advanced metastatic stage. Therefore, exploring the mechanisms underlying metastasis is key to understanding the development of NSCLC. The expression of B cell receptor-associated protein 31 (BCAP31), calreticulin, glucose-regulated protein 78, and glucose-regulated protein 94 were analyzed using immunohistochemical staining of 360 NSCLC patients. It resulted that the high-level expression of the four proteins, but particularly BCAP31, predicted inferior overall survival. What’s more, BCAP31 was closely associated with histological grade and p53 status, which was verified by seven cohorts of NSCLC transcript microarray datasets. Then, three NSCLC cell lines were transfected to observe behavior changes BCAP31 caused, we found the fluctuation of BCAP31 significantly influenced the migration, invasion of NSCLC cells. To identify the pathway utilized by BCAP31, Gene Set Enrichment Analysis was firstly performed, showing Akt/m-TOR/p70S6K pathway was the significant one, which was verified by immunofluorescence, kinase phosphorylation and cellular behavioral observations. Finally, the data of label-free mass spectroscopy implied that BCAP31 plays a role in a fundamental biological process. This study provides the first demonstration of BCAP31 as a novel prognostic factor related to metastasis and suggests a new therapeutic strategy for NSCLC.

## Introduction

Lung cancer is one of the most prevalent neoplasms and the leading cause of cancer-related death worldwide, being responsible for nearly one in five cases^1^. Non-small-cell lung cancer (NSCLC) contributes to 85% of lung cancer cases. Owing to the absence of clinical symptoms and effective screening programs, most lung cancers are diagnosed at an advanced stage with metastasis. Targeted immunotherapy, including anti-angiogenic and checkpoint monoclonal antibodies or tyrosine kinase inhibitors, demonstrates better efficacy than traditional surgical treatment and radio-chemotherapy, although drug resistance and tumor heterogeneity remain significant problems, and metastasis remains the major cause of mortality^2^.

It is therefore important to identify efficient symbolic markers of metastasis and therapeutic targets for NSCLC. Given the aberrant expression of specific genes in a variety of cancer types, restricted in testis or selected in normal tissue, cancer-testis antigens (CTAs) have emerged as efficient specific tumor targets which spare normal tissue from incurring damage during treatment^3^. Originally described in patients with malignant melanoma^4^, CTAs have been identified as biomarkers for a diverse range of cancers, including NSCLC^5^. Their expression is often coordinated^6^, and associated with poor clinical outcome^7^ and advanced stage^8^, particularly metastasis^9^. Some CTAs have already been used as biomarkers for the diagnosis and prognosis of NSCLC, or as targets in clinical trials for vaccine immunotherapy^10^. According to our previous studies^11^, B cell receptor-associated protein 31 (BAP31/BCAP31) is a CTA-like protein which is highly expressed in many forms of cancer, including lung cancer.

*BCAP31*, located on chromosome Xq28, encodes a 28 kDa polytopic integral protein of the endoplasmic reticulum (ER)^12^. As an evolutionarily conserved molecule, the BCAP31 protein contains three predicted transmembrane segments within its amino terminus^12^, and has been implicated in the sorting of a diverse range of ER membrane proteins, as well as participating in the transportation of various molecules from the ER to the Golgi apparatus^13^. As a substrate of caspase, BCAP31 participates in the crosstalk between ER and mitochondria to regulate apoptosis^14^. After being defined as a novel CTA-like protein, BCAP31 has been correlated with hepatocellular carcinoma and breast cancer^15,16^. However, the relationship between BCAP31 and the development of NSCLC remains unclear. It has been reported that ER proteins can influence cell growth, migration, and invasion through epithelial-mesenchymal transition (EMT), ER stress, and autophagy^17–19^. We therefore hypothesized that BCAP31, as an ER chaperone, may play a role in NSCLC metastasis.

Thus far, studies have failed to identify a precise mechanism by which BCAP31 can regulate NSCLC cells. To validate our hypothesis, the present study began with an evidence-based medical evaluation and exploration of the molecular mechanisms of BCAP31. To the best of our knowledge, this is the first study to systematically investigate the significance and biological function of BCAP31, and provides new understanding of NSCLC development and metastasis.

## Results

### BCAP31 protein expression correlates with NSCLC

**Figure 1:**
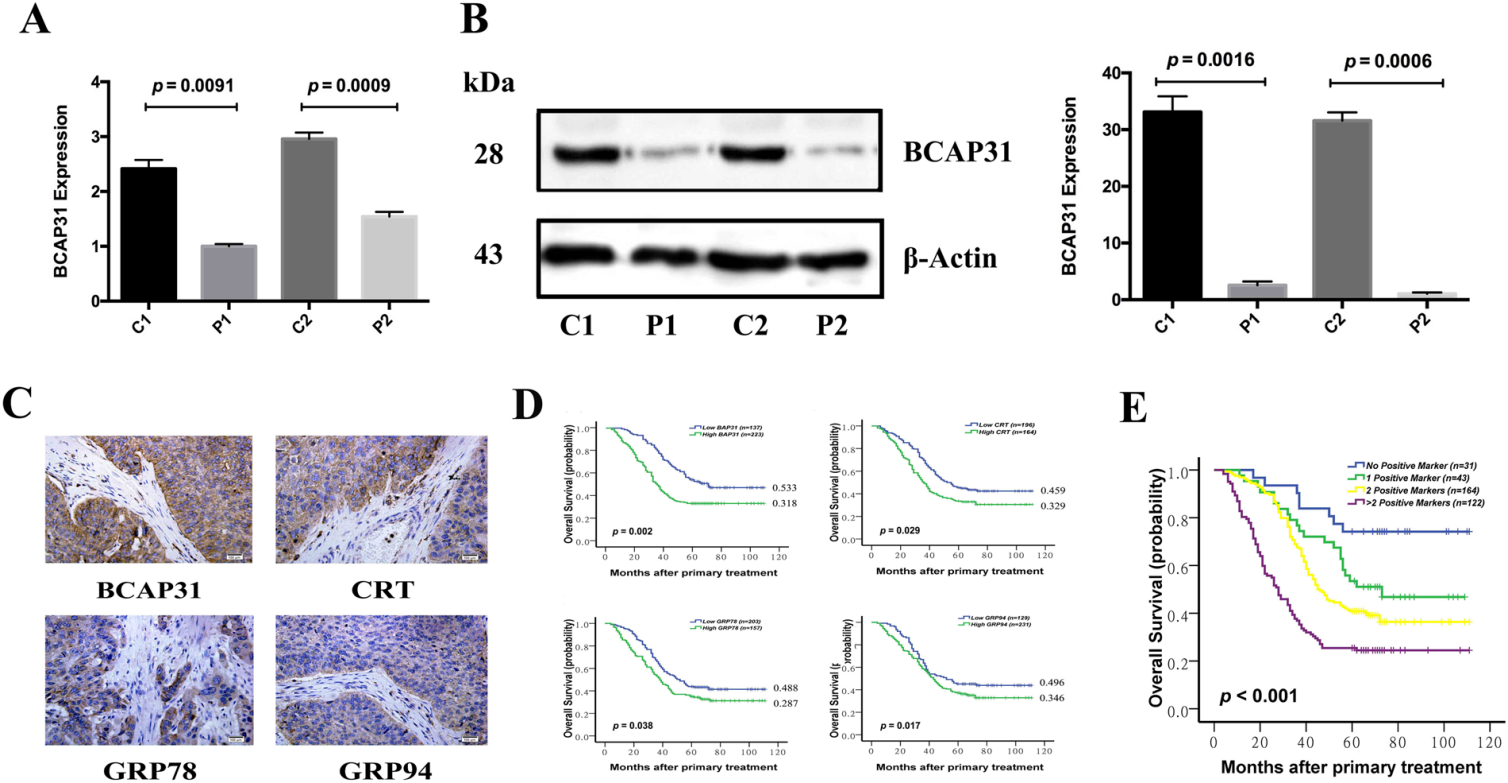
B cell receptor-associated protein 31 (BCAP31) expression in NSCLC and para-carcinoma tissue. **(A)** RT-PCR analysis of two pairs of NSCLC cancerous tissue samples (C1, C2) and their respective para-carcinoma tissues (P1, P2). (**B**) Western blot analysis of two pairs of NSCLC cancerous tissue samples (C1, C2) and their respective para-carcinoma tissues (P1, P2). β-actin was chosen as the reference protein for western blotting because of three reports from the Gene Set Enrichment Analysis hinting that tubulin could be affected by BCAP31 (Supplementary Table S2 sheets 1–3). (**C**) Immunohistochemical analysis for BCAP31, CRT, GRP78, and GRP94 performed on tissues from 360 NSCLC patients. (**D**) Overall survival curves showing the relevance of the expression of four key protein markers and NSCLC patient overall survival. (**E**) The presence of the four markers alone, or in combination, resulted in distinct differences in the overall survival of NSCLC patients. C: cancer tissue; P: para-carcinoma tissue. All experiments were repeated at least three times.

First, we investigated the gene and protein expression of BCAP31. Compared to adjacent tissues, NSCLC tissues had higher levels of both BCAP31 gene and protein expression, as assessed using qRT-PCR and western blot analysis (Fig. 1A,B). To investigate the clinical significance of BCAP31 in NSCLC, we examined the expression of three well-known tumor markers, calreticulin (CRT), glucose-regulated protein 78 (GRP78) and glucose-regulated protein 94 (GRP94), as parallel control. The expression of the four proteins was analyzed immunohistochemically using a tissue microarray of samples from 360 NSCLC patients and16 normal controls. Of the elevated levels of the four proteins (Fig. 1C), BCAP31 was significantly associated with histological grade (*p* = 0.017) (Table 1). At the time of analysis, 137 of the 360 patients were still alive, with a median follow-up of 78 months. Kaplan–Meier survival curves were plotted and Fisher’s exact probability test was performed, which indicated that high expression was associated with patient overall survival (Fig. 1D, Table 2). Retrospective χ^2^ tests demonstrated that clinicopathological stage and histological grade were risk factors for cancer-related death. Multivariate Cox analysis further demonstrated an enhanced survival prediction of their synergetic effect (Fig. 1E), which implied that BCAP31 could represent a new prognostic factor. Analysis of seven transcript expression microarray datasets extracted from the Gene Expression Omnibus (GEO) (https://www.ncbi.nlm.nih.gov/geo/) validated the positive correlation of BCAP31 expression with histological grade (*p* = 0.0009) and p53 status (*p* = 0.0058; Supplementary Table S1). However, there was no correlation between BCAP31 mRNA expression and NSCLC prognosis, which was inconsistent with our protein analysis (Supplementary Fig. S1).

**Table 1:** Patient Demographics (n = 360).

Characteristic	All Patients	BCAP31 Expression			CRT Expression			GRP78 Expression			GRP94 Expression		
		High (n = 223)	Low (n = 137)	*P*	High (n = 164)	Low (n = 196)	*P*	High (n = 157)	Low (n = 203)	*P*	High (n = 231)	Low (n = 129)	*P*
Age, years				0.161			0.328			0.35			0.595
Median	58.5	59.6	56.7		59.3	57.9		59.2	58.0		58.8	58.4	
Range	28–77	25–77	28–75		34–77	25–75		34–76	25–77		25–76	37–77	
Histological type				0.992			0.348			0.811			0.975
Squamous cell carcinoma	239	148	91		109	130		104	135		157	82	
Adenocarcinoma	85	53	32		35	50		40	45		56	29	
Large cell carcinoma	36	22	14		20	16		17	19		23	13	
Clinico-pathologic stage				0.373			0.114			0.007			0.773
I (Ia + Ib)	139	80	59		54	85		44	95		90	49	
II (IIa + IIb)	131	86	45		67	64		66	65		89	42	
III (IIIa + IIIb)	90	57	33		43	47		39	51		62	28	
Tumor size, cm				0.725			0.052			0.185			0.071
<3	35	20	15		10	25		19	16		21	14	
3–7	276	174	102		127	149		112	164		182	94	
>7	49	29	20		27	22		17	32		24	25	
Histological grade				0.017			0.23			0.484			0.543
High (grade 1)	77	47	30		29	48		33	44		52	25	
Moderate (grade 2)	164	91	73		73	91		71	93		99	65	
Poor (grade 3)	83	64	20		42	41		44	39		57	26	
Unidentified	36	22	14		20	16		17	19		23	13	
Nodal status				0.588			0.848			0.25			0.212
N0	198	121	77		94	104		79	119		120	78	
N1	97	64	33		43	54		45	52		64	33	
N2	65	38	27		29	36		33	32		47	18

**Table 2:** Standard Clinicopathologic Variables, BCAP31, CRT, GRP78 and GRP94 Expression Related to Survival.

Variable	No. of Patients	No. of Deaths	% Survival at 5 Years	*P*
Age, years				0.292
<55	149	90	41.6	
55–65	113	66	43.4	
>65	98	67	33.7	
Histological type				0.066
Squamous cell carcinoma	239	148	40.2	
Adenocarcinoma	85	47	44.7	
Large cell carcinoma	36	28	27.8	
Clinico-pathologic stage				0.005
I (Ia + Ib)	139	73	50.4	
II (IIa + IIb)	131	84	38.2	
III (IIIa + IIIb)	90	66	26.7	
Tumor size, cm				0.039
<3	35	23	37.1	
3–7	276	162	43.5	
>7	49	38	22.4	
Histological grade				<0.001
High (grade 1)	77	39	51.9	
Moderate (grade 2)	164	92	45.7	
Poor (grade 3)	83	64	22.9	
Unidentified	36	28	27.8	
Nodal status				0.142
N0	198	116	44.4	
N1	97	60	39.2	
N2	65	47	27.7	
BCAP31 Exptession				0.002
High	223	152	31.8	
Low	137	71	53.3	
CRT Exptession				0.029
High	164	112	32.9	
Low	196	111	45.9	
GRP78 Exptession				0.038
High	157	116	28.7	
Low	203	107	48.8	
GRP94 Exptession				0.031
High	231	154	34.6	
Low	129	69	49.6	

### BCAP31 promotes NSCLC cell migration and invasion

**Figure 2:**
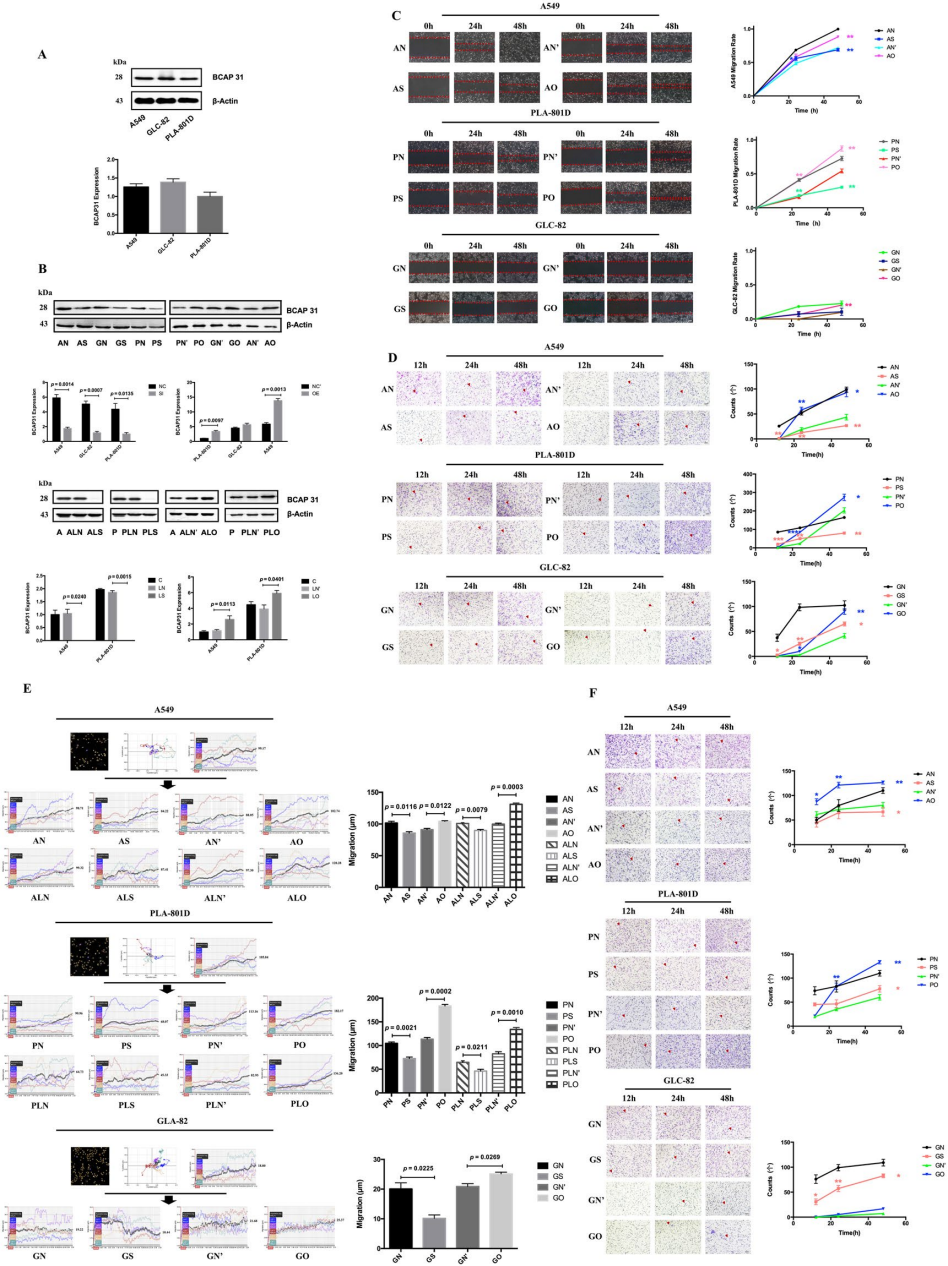
The effects of BCAP31 on the migration and invasion of three NSCLC cell types. (**A**) Western blot analysis of BCAP31 expression in A549, GLC-82, and PLA-801D cells. (**B**) Western blot analysis of BCAP31 expression in NSCLC cells transiently and stably transfected using siRNA, plasmid, or lentivirus. (**C**) Wound-healing experiments showed that in transiently transfected cells, decreased BCAP31 expression(AS, PS, GS) hindered the activity of cells compared to controls(AN, PN, GN) after 24 h(the experimental groups and respective controls were analyzed using a *t* test; differences shown are statistically significant when *p* < 0.05. Significant differences: p_AN, AS_ = 0.0328, p_PN, PS_ = 0.0014 at 24 h; and p_AN, AS_ = 0.0045, p_PN, PS_ = 0.0034 at 48 h), while the groups with increased expression(AO, PO, GO) showed the opposite trend compared to their controls(AN’, PN’, GN’)(significant differences: p_PN’, PO_ = 0.0032 at 24 h; and p_AN’, AO_ = 0.0014, p_PN’, PO_ = 0.0031, and p_GN’, GO_ = 0.0030 at 48 h). (**D**) Transwell migration experiments showed the same trend as the wound-healing tests in transiently transfected cells(data were analyzed using a *t* test; differences shown are statistically significant when *p* < 0.05). (**E**) In one field of view, five cells travelling in different directions were randomly chosen and monitored for 96 h. The HoloMonitor M4 recorded all cell indices (migration, area, optical thickness, and track) and showed the same migration trend as the above experiments, regardless of whether cells were transiently or stably transfected(data were analyzed using a *t* test; differences shown are statistically significant when *p* < 0.05). (**F**) Transwell invasion experiments showed the same trend as the above experiments in transiently transfected cells(data were analyzed using a *t* test; differences shown are sta*t*istically significant when *p* < 0.05). A: A549; P: PLA-801D; G: GLC-82; AN, PN, GN/AS, PS, GS: control/experimental groups of decreased BCAP31 expression with transient transfectio; AN’, PN’, GN’/AO, PO, GO: control/experimental groups of increased BCAP31 respectively; ALN, PLN/ALS, PLS: control/experimental groups of decreased BCAP31 expression with stable transfection; ALN’, PLN’/ALO, PLO: control/experimental groups of increased BCAP31 respectively. All experiments were repeated at least three times.

To investigate the role of BCAP31 in NSCLC cells, its expression was first detected in three cell types (A549, GLC-82, and PLA-801D) (Fig. 2A). Cells were transiently transfected using siRNA and plasmid (in A549, GLC-82, and PLA-801D), and lentivirus for stable transfection (in A549 and PLA-801D) to achieve reduced or increased levels of expression of BCAP31 for subsequent experiments. Specifically, for transiently transfected cells, AS, GS, and PS were transfected using BCAP31 siRNA and Lipofectamine 3000, and the cells expressed lower levels of BCAP31 than controls (AN, GN, PN), whereas AO, GO, PO were transfected using BCAP31 plasmid and Lipofectamine 3000, and the cells expressed higher levels of BCAP31 than controls (AN’, GN’, PN’). For stably transfected cells, lentiviral vectors were used, and ALS and PLS transfection resulted in significant decreases in BCAP31 expression compared to controls (ALN, PLN) while ALO and PLO transfection resulted in increased BCAP31 expression compared to controls (ALN’, PLN’). All transfections were successful (Fig. 2B, Supplementary Fig. S1B). Depletion of BCAP31 inhibited the wound-healing activity (Fig. 2C) and reduced migration (Fig. 2D,E, Supplementary Vids. S1–S23) and invasion (Fig. 2F) of all cell lines, compared to the negative control. A contrasting effect emerged upon BCAP31 elevation, which implied that BCAP31 might affect the development of NSCLC.

### BCAP31 caused minor changes in cellular morphology and F-actin distribution

**Figure 3:**
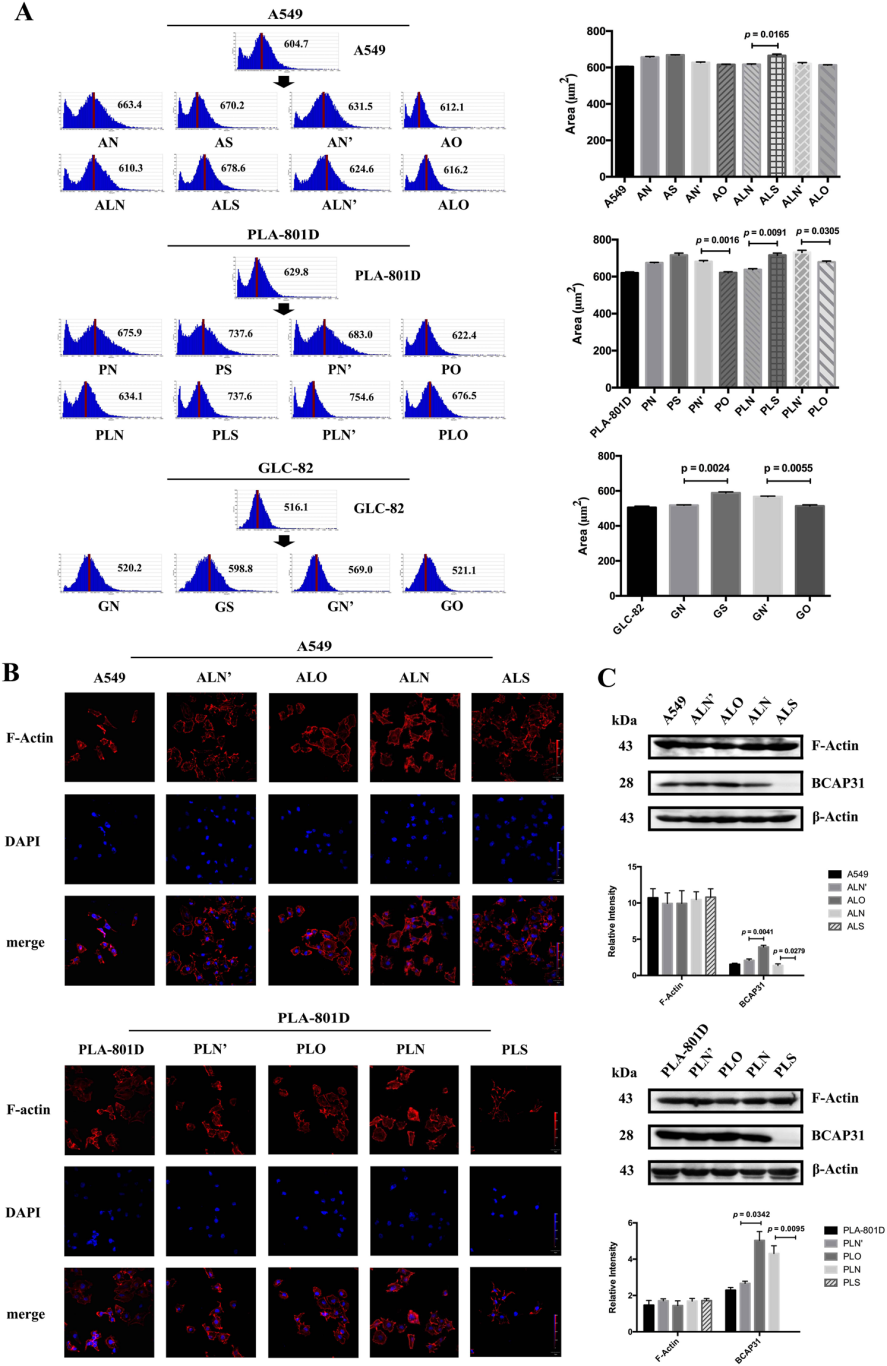
BCAP31 could induce minor changes in cellular morphology and the F-actin distribution in NSCLC cells. (**A**) During HoloMonitor M4 area recording of NSCLC cells, groups with lower level of BCAP31 showed larger areas than controls, while groups with higher level of BCAP31 were the opposite. A *t* test was used for the analysis of each group. Significant differences: *p*_ALN, ALS_ = 0.0165, *p*_PN’, PO_ = 0.0016, *p*_PLN, PLS_ = 0.0091, *p*_PLN’, PLO_ = 0.0305, *p*_GN, GS_ = 0.0024, *p*_GN’, GO_ = 0.0055. (**B**) An immunofluorescence assay showed that a mildly clearer cell outline could be seen in the BCAP31-rich cells of each group (ALO, PLO, ALN, PLN) than in BCAP31-depleted cells (ALN’, PLN’, ALS, PLS), which means that F-actin distribution changed a little after BCAP31 was modulated. (**C**) Western blot analysis of F-actin expression showed stable levels, regardless of BCAP31 modulation. AN, PN, GN: control groups of decreased BCAP31 expression with transient transfection in three cell lines; AS, PS, GS: experimental groups of decreased BCAP31 expression with transient transfection in three cell lines; AN’, PN’, GN’: control groups of increased BCAP31 expression with transient transfection in three cell lines; AO, PO, GO: experimental groups of increased BCAP31 expression with transient transfection in three cell lines; ALN, PLN: control groups of decreased BCAP31 expression with stable transfection in two cell lines; ALS, PLS: experimental groups of decreased BCAP31 expression with stable transfection in two cell lines; ALN’, PLN’: control groups of increased BCAP31 expression with stable transfection in two cell lines; ALO, PLO: experimental groups of increased BCAP31 expression stable transfection in two cell lines. All experiments were repeated at least three times.

To gain a better view of cell morphological changes, HoloMonitor M4 was used to collect real-time images of unlabeled live cells using a low-power 635 nm diode laser. After transfection, regardless of whether BCAP31 expression was up- or down-regulated, all cells showed a change in size. When BCAP31 expression was decreased, cells from some groups (A549, PLA-801D, and GLC-82) were larger than the negative control, while some cells (PLA-801D and GLC-82) were smaller than the negative control, with increased BCAP31 expression (Fig. 3A).

A GSEA report, including 315 significant gene sets, showed that cytoskeletal protein and mRNA levels were influenced by BCAP31 changes (Supplementary Table S2 sheet 4). To confirm this, firstly, an immunofluorescence assay was performed which showed that transfected cells had slightly larger cell areas than normal cells, and that BCAP31 over-expression produced mildly clearer cell outlines, implying that a little more F-actin was expressed at the periphery; low expression levels resulted in the opposite effect (Fig. 3B). We collected stably transfected A549 and PLA-801D cell lines and analyzed their F-actin protein levels, which turned out to be independent of BCAP31 expression (Fig. 3C). Taken together, BCAP31 may have had a marginal impact on the distribution rather than the expression of F-actin, which could have resulted in small changes in cell migration.

### BCAP31 did not significantly affect NSCLC cell cycle and apoptosis

Several Gene Set Enrichment Analysis (GSEA) reports on data from the Kyoto Encyclopedia of Genes and Genomes, Reactome, and BioCarta databases demonstrated the potential involvement of BCAP31 in cell cycle and apoptosis (Supplementary Fig. S2A). To verify this effect, flow cytometric analysis was performed, which revealed that BCAP31 did not significantly affect cell cycle and apoptosis (Supplementary Fig. S2B,C). In addition, we analyzed clone formation and monitored cell proliferation using the xCELLigence RTCA DP instrument. It emerged that up- and down-regulation of BCAP31 caused only minor inhibition to cell proliferation (Supplementary Fig. S2D,E), which suggested that BCAP31 dysregulation may lead to disorders of cell multiplication. Also, according to RTCA results, there was little fluctuation in the cell irregularity data, implying that all cells were in good condition and that each cell strain demonstrated its own irregularity (Supplementary Fig. S2F). Finally, the optical thickness of cells showed no consistent change in the three cell lines (Supplementary Fig. S2G).

### BCAP31 was not related to EMT in NSCLC metastasis

**Figure 4:**
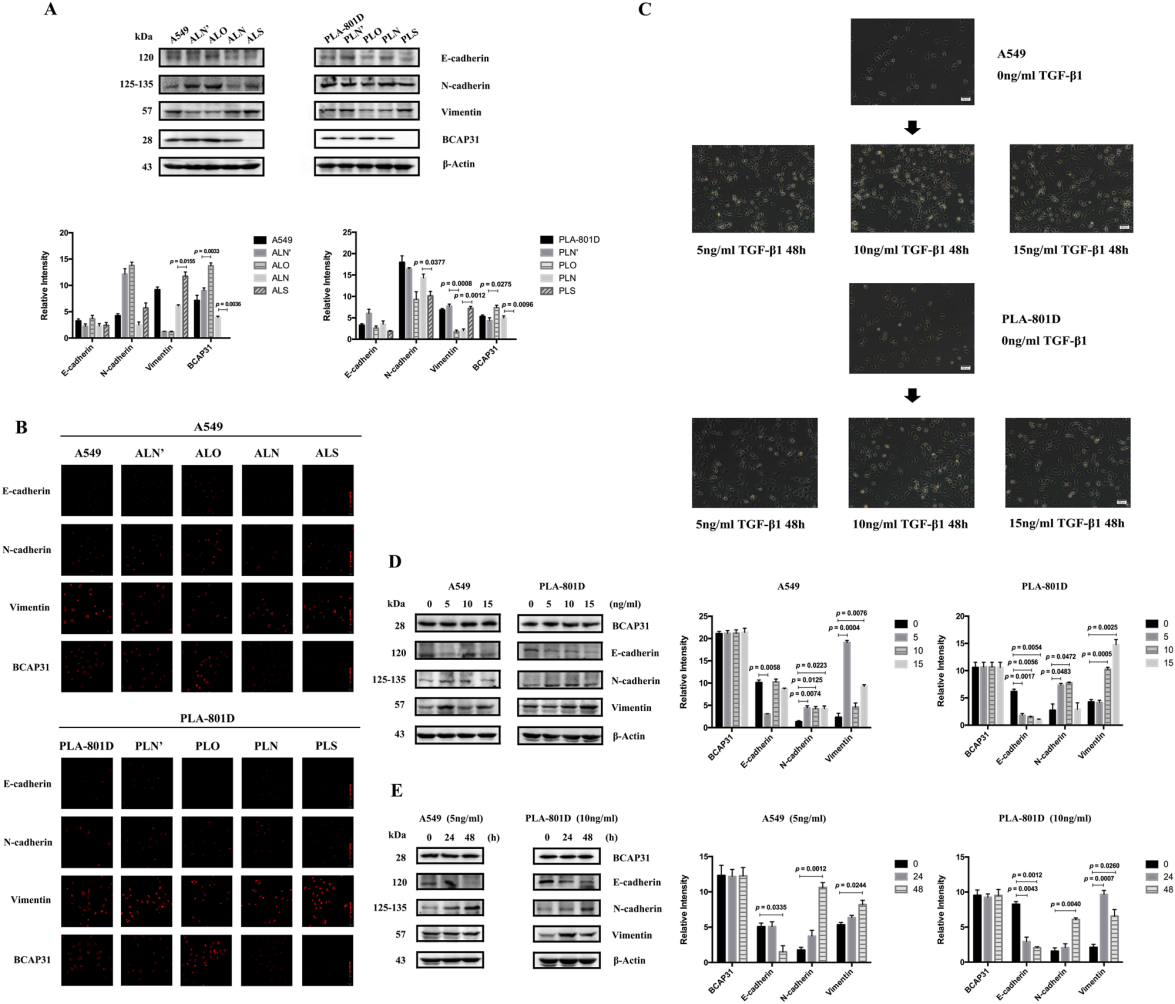
BCAP31 is not related to EMT. (**A**) Western blot analysis of the main EMT marker proteins showed that when BCAP31 was down-regulated in A549 cells, levels of the epithelial marker E-cadherin and mesenchymal markers vimentin and N-cadherin increased compared to negative controls. N-cadherin and E-cadherin expression increased when BCAP31 was increased in A549 cells, while vimentin expression remained unchanged. In PLA-801D cells, the expression of the three proteins decreased with high BCAP31 expression, and N-cadherin and E-cadherin expression were reduced; vimentin expression increased with low BCAP31 expression. (**B**) Immunofluorescence assays corroborated the results shown in (A). (**C**) Images captured under a light microscope showing that after stimulation using different concentrations of TGF-β1 for 48 h, some cells became spindle-shaped and cell–cell adhesion decreased. (**D**) Western blot analysis following stimulation using different concentrations of TGF-β1 for 48 h. EMT was efficiently induced in which E-cadherin expression decreased, and N-cadherin and vimentin expression significantly increased at 5 ng/mL for A549 cells, and 10 ng/mL for PLA-801D cells. Under these conditions, BCAP31 expression remained unchanged. (**E**) Western blot analysis at different times following stimulation using appropriate concentrations of TGF-β1. EMT was efficiently induced after 48 h. Under these conditions, BCAP31 expression was unchanged. ALN, PLN: control groups of decreased BCAP31 expression with stable transfection in two cell lines; ALS, PLS: experimental groups of decreased BCAP31 expression with stable transfection in two cell lines; ALN’, PLN’: control groups of increased BCAP31 expression with stable transfection in two cell lines; ALO, PLO: experimental groups of increased BCAP31 expression stable transfection in two cell lines. All experiments were repeated at least three times.

In order to identify the mechanism underlying how BCAP31 promotes cell migration and invasion, we evaluated markers of EMT in transfected cells. Western blotting and immunofluorescence experiments were negative (Fig. 4A,B). Furthermore, transforming growth factor-β1 (TGF-β1) stimulation was performed (Fig. 4C), and BCAP31 expression was shown to be irrelevant to the induced EMT process (Fig. 4D,E).

### The Akt/mTOR/p70S6K pathway is BCAP31-dependent in affecting NSCLC metastasis

**Figure 5:**
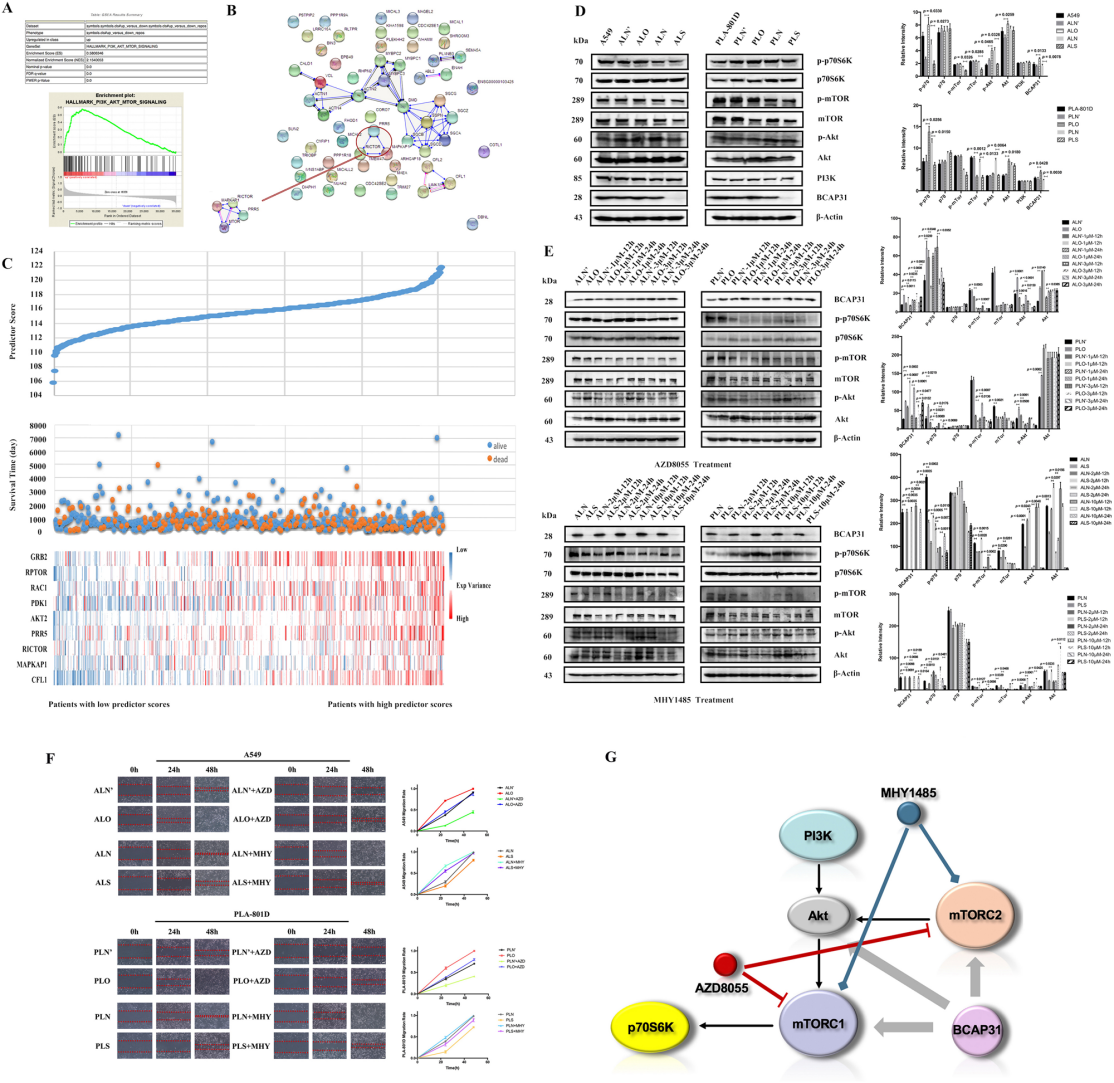
BCAP31 is involved in the Akt/mTOR/p70S6K pathway and influences NSCLC metastasis. (**A**) GSEA database prediction showed that BCAP31 may be involved in the PI3K/Akt/mTOR pathway. (**B**) Genes which are relevant to F-actin were selected from the STRING database. (**C**) The relationship between the expression of nine genes and patient death risk. (**D**) The participation of BCAP31 in the Akt/mTOR/p70S6K pathway, except for PI3K, was verified by western blotting in stably transfected cells featuring the up- and down-regulation of BCAP31. (**E**) Western blot analysis of the main proteins in the PI3K/Akt/mTOR/p70S6K pathway after using the mTOR inhibitor AZD8055 and the mTOR agonist MHY1485. 12 h stimulation with 1 μM (for A549, PLA-801D cells) and 3 μM (for A549 cells) of AZD8055, as well as 2 μM (for A549, PLA-801D cells) and 10 μM (for PLA-801D cells) of MHY1485, showed that BCAP31 expression was unaffected under a successful drug effect. (**F**) Wound-healing experiments were performed using stably transfected cells with or without AZD8055 or MHY1485. Regardless of the presence of AZD8055, cells over-expressing BCAP31 migrated faster than controls, but the use of AZD8055 slowed down the pace of this migration. A *t* test was used for analysis of each group. Similarly, regardless of the presence of MHY1485, BCAP31 knock-down cells migrated slower than controls, but the use of MHY1485 increased the pace of this migration. A *t* test was used for analysis of each group. (**G**) The relationships between the PI3K/Akt/mTOR/p70S6K pathway, BCAP31, AZD8055 and MHY1485. Akt, mTORC1 and mTORC2 were dependent on BCAP31 expression. AZD8055 inhibited mTORC1 and mTORC2 whereas MHY1485 produced the opposite effect. All experiments were repeated at least three times.

The PI3K/Akt/mTOR pathway gene set was significantly enriched under the condition of BCAP31 differential expression (Fig. 5A). Given that BCAP31 could slightly influence F-actin distribution, genes relevant to F-actin were investigated in the STRING database. We found that proline-rich protein 5 (*PRR5*), mitogen-activated protein kinase associated protein 1 (*MAPKAP1*), and RPTOR-independent companion of mTOR complex 2 (*RICTOR*) (all of which can interact with *mTOR*) were included in the resultant list (Fig. 5B). Nine genes, including growth factor receptor bound protein 2 (*GRB2*), regulatory associated protein of mTOR complex 1 (*RPTOR*), Rac family small GTPase 1 (*RAC1*), pyruvate dehydrogenase kinase 1 (*PDK1*), AKT serine/threonine kinase 2 (*AKT2*), *PRR5, RICTOR, MAPKAP1* and cofilin 1 (*CFL1*) of the PI3K/Akt/mTOR pathway were identified to potentially have significant interaction with BCAP31 according to GSEA prediction. Multivariate Cox regression analysis of transcriptomic data from 506 patients with lung adenocarcinomas (LUAD) from The Cancer Genome Atlas (TCGA) suggested that the high expression of these nine genes served as a good adverse prognostic predictor of NSCLC (Fig. 5C). Then, Western blot analysis of the main proteins in the PI3K/Akt/mTOR/p70S6K pathway, showing that when BCAP31 expression was decreased, PI3K and p70S6K expression remained stable, and levels of Akt, p-Akt, mTOR, p-mTOR and p-p70S6K expression all fell compared to the control group, which was consistent in both A549 and PLA-801D. However, when BCAP31 expression was increased in A549 cells, PI3K and p70S6K expression still remained stable, Akt, p-Akt and p-p70S6K expression increased, while mTOR and p-mTOR slightly improved. As for PLA-801D cells, when BCAP31 expression was increased, PI3K and p70S6K expression remained the same, Akt, p-Akt and p-p70S6K expression increased, while mTOR expression decreased and p-mTOR expression slightly increased (Fig. 5D). In addition, after 12 h of stimulation using 1 μM and 3 μM AZD8055 in A549 cells, p-Akt, p-mTOR and p-p70S6K expression decreased, while expression of Akt, mTOR and p70S6K remained stable; this resulted in the successful inhibition of the pathway, but had no influence on BCAP31. The same happened in PLA-801D cells following incubation with 1 μM AZD8055 for 12 h. Similarly, effective mTOR pathway activation was triggered after stimulation with 2 μM MHY1485 for 12 h in A549 cells and resulted in a distinct elevation of Akt, p-Akt, p-mTOR and p-p70S6K expression compared to the control. In PLA-801D cells, there was a consistent increase in Akt, p-Akt, p-mTOR and p-p70S6K expression following a 12 h incubation with 2 μM and 10 μM MHY1485. However, levels of BCAP31 were not affected (Fig. 5E). Furthermore, the responses of a BCAP31 differentially expressed model to such stimulation were mildly different, which suggested heterogeneity between tumor cells. The finding was also confirmed by a wound-healing assay with stably transfected cells (Fig. 5F).

## Discussion

In the present study, we firstly revealed the clinical importance of BCAP31 in NSCLC, and that it was closely associated with cancer development. BCAP31 expression was higher in cancerous tissue than adjacent tissues at both mRNA and protein levels. This level of expression was consistent with a CTA pattern, indicating that BCAP31 represents a promising therapeutic target. BCAP31, in parallel with the other three markers, was also identified as a useful prognostic factor for NSCLC, as demonstrated by immunohistochemical staining. All four proteins showed statistical significance; however, the differential expression of BCAP31 was more associated with cancer malignancy, development, and the longest median overall survival. Clinicopathological stage and histological grade were associated with GRP78 and BCAP31, respectively (Tables 1, 2). This phenomenon for GRP78 was familiar to us^20^; however, this was the first time that BCAP31 has been associated with the differentiation and malignancy of NSCLC, which may be due to BCAP31 exhibiting stemness functionality^21^. Survival prediction efficiency of NSCLC patients improved as more markers were included, suggesting that BCAP31 might play a similar role to the other three markers in promoting cancer metastasis^22,23^.

The migration and invasion of tumor cells mainly relies on factors such as enhanced mobility^24^, depressed intercellular adhesion and the degradation of extracellular matrix^25^. BCAP31 promoted NSCLC cell motility and migration in wound-healing assays, transwell assays without matrigel, and HoloMonitor M4 monitoring migration. On the other hand, transwell assays with matrigel demonstrated that BCAP31 promoted cell migration through the extracellular matrix. EMT was verified by western blotting; the expression of BCAP31 did not influence EMT, while TGF-β1-induced EMT was not related to the expression of BCAP31 protein. The role of EMT in metastasis is a long-standing controversy, largely because of the inability to monitor transient and reversible EMT phenotypes *in vivo*. Without evidence for the dissemination, colonization, and metastatic outgrowth of mesenchymal tumor cells, the role of EMT will remain contested.

Previous studies have shown that the Akt/mTOR pathway is activated in many lung cancer patients^26^. This signaling pathway could activate p70S6k to promote actin filament reconstruction to enhance cell migration^27^ and induced hypoxia-inducible factor-1 production, which results in cancer metastasis. In addition, this pathway could increase the mRNA and protein expression of matrix metalloproteinase-2 with which to degrade the extracellular matrix and thus facilitate cell migration and invasion^28^. It could also facilitate tumor metastasis by regulating endothelial nitric oxide synthase, nuclear factor kappa-light-chain-enhancer of activated B cells, and epidermal growth factor receptors^29^. Furthermore, one of the mTOR signaling complexes, mTORC2, can promote cell migration via the activation of Rho GTPases^30^. In our research, GSEA reports implied that this particular pathway may be affected by BCAP31 expression.

Both HoloMonitor M4 analysis of cell areas, and immunofluorescence staining of F-actin, showed that BCAP31 affected cell morphology and marginally promoted F-actin redistribution to the periphery, which could facilitate cell migration and invasion. STRING analysis further identified F-actin-relevant genes, including *PRR5*, *RICTOR* and *MAPKAP1* (all of which are connected with *MTOR*), which provided further evidence of a role for the Akt/mTOR pathway in cell migration and invasion. Further interpretation of western blotting analysis and wound-healing experiments, and analysis of our previous results, appears to suggest that BCAP31 is not a member of the Akt/mTOR/p70S6K pathway, but can affect the other molecules in this critical pathway (Fig. 5G) in order to influence cell migration.

The Akt/mTOR pathway has also been implicated in the tumorigenesis of NSCLC and apoptosis^31^, and is a central regulator of glycolysis, cancer metabolism, and cancer cell proliferation^32^. mTORC1, another mTOR signaling complex, can promote protein synthesis in response to growth factors and nutrients via the phosphorylation of p70S6K and 4EBP1^30^. Many researchers have also shown that the Akt/mTOR/p70s6K pathway is beneficial for cell proliferation and survival^33^. However, in our studies, either up- or down- regulation of BCAP31 contributed to mildly poor proliferation and irregular effects on cell cycle and apoptosis, which indicated that there may be other pathways involved, in addition to Akt/mTOR/p70s6K, to keep the condition in balance, such as the p53 pathway (Supplementary Table S2 sheet 4). With regards to this point, previous studies have shown that the caspase family was a significant part of the p53 pathway^34^. BCAP31 could regulate procaspase-8L processing^35^ and be cleaved by caspase-8^12^. The cleaved BCAP31 fragment, p20, is a potent inducer of apoptosis when expressed ectopically^36^. Therefore, the enhanced expression of BCAP31 could contribute to the poor survival of NSCLC cells^37^, which should benefit patients. However, full-length BCAP31 inhibits caspase activity^38^, and in cancer, apoptosis-resistance mechanisms are used to evade cell death^39^. Aside from this, the Akt/mTOR/p70s6K pathway also play a significant role in cell autophagy^40^. However, there have been no predictive markers identified as yet that correlate with the clinical activity of novel targeted Akt or dual PI3K/mTOR agents, despite efforts to evaluate tumor molecular status in the early phases of therapeutic clinical trials^41^. Our research identified a new pathway involving BCAP31, although the mechanism by which BCAP31 affects the Akt/mTOR/p70S6K pathway has not been completely characterized, and this should be the aim of future NSCLC metastasis research.

Furthermore, label-free mass spectrometric (MS) analysis for mechanism exploration demonstrated the involvement of BCAP31 in more primitive functions such as translational elongation and ribosome modification, instead of any specific signaling pathway (Supplementary Table S2 sheets 5–6). This implied that BCAP31 plays a role in a fundamental biological process. Combining its CTA characteristics, whereby its expression is restricted to germline and developmental processes, and its downregulation in terminally differentiated tissues and re-expression in cancer, suggest that BCAP31 might bridge fundamental processes with tumorigenesis. This is also consistent with BCAP31 being an ER chaperone, a component of several large protein complexes^42^ that associates with newly synthesized integral membrane proteins and controls their fate.

As an ER chaperone, BCAP31 is thought to regulate protein activation and folding, and thus is essential to other ER proteins^43^. Dysfunction of this ER chaperone could lead to the accumulation of stress signals that activate the unfolded protein response (UPR), endoplasmic reticulum overload response (EOR), and caspase-12 induced pathway to re-establish protein balance in cells undergoing stress^44^. Otherwise, this could promote metastatic progression^45^. Furthermore, the loss of BCAP31 may cause retention of important proteins in the ER^42^. Consequently, ER stress could represent a cause or consequence of various diseases, including cancer^46^, and may play a role in the development and progression of cancer^47^. According to MS analysis and GSEA prediction, many basic biological processes, molecules, and pathways are closely related to BCAP31. Thus, BCAP31 may play a significant role in interfering with NSCLC cell survival by balancing, instead of being beneficial to, tumorigenesis.

The present study confirms, for the first time, that BCAP31 is essential for NSCLC cells to maintain or promote their abnormal proliferation and metastasis, and that the complex relationship between BCAP31, Akt/mTOR/p70S6K, and other pathways, needs to be elucidated in future research studies. These results highlight the potential role for BCAP31 as a prognostic marker and target for NSCLC, as well as yielding mechanistic insights into how BCAP31 regulates NSCLC metastasis.

## Materials and Methods

### Cell culture and treatment

A549 (ATCC), PLA-801D, and GLC-82 cells (held by our laboratory) were all cultured in RPMI 1640 medium (HyClone, Logan, Utah, USA) containing 10% new-born calf serum (Si Ji Qing, Hangzhou, China) and 1% penicillin–streptomycin solution (Corning, Corning, New York, USA). 5 ng/ml, 10 ng/ml, 15 ng/ml recombinant human TGF-β1 (PeproTech, Rocky Hill, New Jersey, USA) were used for 48 h to induce EMT, followed by Western blot analysis. AZD8055 and MHY1485 (Selleck, Houston, Texas, USA) were used to affect mTOR pathway, followed by Western blot analysis.

### Tissue specimens

16 paraffin-embedded normal tissue samples and tumor samples from 360 NSCLC patients (Chaoying, Xi’an, China), obtained between January 2002 and March 2006 from the Xijing Hospital, the Fourth Military Medical University, were analyzed. Clinico-pathological data were obtained from all patients via a retrospective review of medical charts. The clinico-pathological staging of tumors was classified according to the 7th edition of the TNM classification system for lung cancer^48^. The retrospective study of tumor samples was approved by the Ethics Committee of the Fourth Military Medical University, and written informed consent was obtained from each patient. The clinico-pathological characteristics of the patients involved in our study are presented in Table 2.

### Real-time quantitative reverse-transcription PCR (qRT-PCR) assay

Total RNA was extracted using TRIzol RNA Isolation Reagents (Invitrogen, Carlsbad, New Mexico, USA) and reverse-transcribed using the HiScript II Q Select RT SuperMix for qPCR (Vazyme, Nanjing, China). PCR primers for BCAP31 were purchased from Sangon Biotech (Shanghai, China); targeting sequences were 5′-CGGCTGGTGGAGTTGTTAGT-3′ (sense) and 5′-CGGGATTGTTCTGGAGGTT-3′ (antisense). Data shown are representative of at least three experiments with similar results, unless otherwise mentioned.

### Western blot analysis

Cells and tissues were extracted in Radio-Immunoprecipitation Assay (RIPA) buffer (Vazyme, Nanjing, China) with phenylmethylsulfonyl fluoride (PMSF) (Genshare Biological, Xi’an, China). Proteins were then separated using 10% sodium dodecyl sulfate polyacrylamide gel electrophoresis (Genshare Biological, Xi’an, China) and transferred onto a nitrocellulose membrane. Membranes were blocked using 5% milk (BD, Franklin Lake, New Jersey, USA) for 1 h, and proteins were probed using primary antibodies against the following proteins: β-actin, vimentin (Proteintech, Wuhan, China), BCAP31, N-cadherin, E-cadherin (Abcam, Cambridge, UK), F-actin (Bioss, Beijing, China), PI3K, Akt, p-Akt, mTOR, p-mTOR, p70S6K and p-p70S6K (CST, Boston, Massachusetts, USA). Primary antibodies were incubated at 4 °C overnight and horseradish peroxidase (HRP)-conjugated secondary antibodies (Proteintech, Wuhan, China) were incubated at room temperature for 1 h to allow visualization of positive signals.

### Plasmid construction

First, the coding sequence of the *BCAP31* gene was synthesized (gene ID:10134, NCBI Reference Sequence: NM_005745.7 for overexpression and NM_001139457 for knock-down) (using the green fluorescence protein (*GFP*) gene as a reporter gene and the *Puro* gene as a resistant gene). The plasmids were constructed by GeneCreate (Wuhan, China) and Genechem (Shanghai, China).

### Lentivirus package

The lentiviral expressing and packaging plasmid mix were extracted using an EndoFree maxi plasmid kit (Tiangen, Beijing, China). Plasmid DNA and Lipofectamine 2000 reagent (Invitrogen, Carlsbad, USA) were mixed in serum-free medium and used to transfect 293T cells (ATCC, Rockefeller, Maryland, USA). After 6–10 h, the transfection solution was changed to Dulbecco’s modified Eagle’s medium (HyClone, Logan, USA) containing 10% fetal bovine serum (FBS) (Si Ji Qing, Hangzhou, China). The supernatant was collected after 48 h. The virus titer was then determined using fluorescence dilution methods.

### Lentivirus infection and cell selection

A549, PLA-801D and GLC-82 cells were seeded in 96-well plates. After 24 h, 10 μL of virus (diluted in enhanced infection solution [ENi.S.], 1 × 10^7^ TU/mL) was added to 80 μL of ENi.S. and 10 μL of polybrene (E) (diluted polybrene in ENi.S., 50 μg/mL). After 12 h, the infection solution was changed to fresh nutrient medium. Puromycin (5 μg/mL) (MP Biomedicals, Shanghai, China) was added into the supernatant for 1 week to select for transfected cells. GLC-82 cell lines with stably up-regulated BCAP31 can’t established.

### siRNA interference and transfection

BCAP31-siRNA was purchased from GenePharma (Shanghai, China); the targeting sequences were 5′-GGUGAACCUCCAGAACAAUTT-3′ (sense) and 5′-AUUGUUCUGGAGGUUCACCTT-3′ (antisense) and negative control siRNA sequences were 5′-UUCUCCGAACGUGUCACGUTT-3′ (sense) and 5′-ACGUGACACGUUCGGAGAATT-3′ (antisense). All transient transfection was performed using Lipofectamine 3000 reagent (Invitrogen, Carlsbad, New Mexico, USA) for 48 h.

### xCELLigence real-time cellular analysis (RTCA)

Cell proliferation was recorded using the xCELLigence RTCA DP instrument (Roche Diagnostics GmbH, Mannheim, Germany) in modified 16-well plates (E-plate, Roche Diagnostics GmbH). Plates were locked into the RTCA DP device in an incubator and the impedance value was automatically monitored and expressed as a cell index value (CI) every 15 min for 90 h. All data were recorded using Real Time Cellular Analysis (RTCA) software (version 1.2.1).

### Clone formation assay

Cells were collected during the logarithmic growth phase and seeded at 100, 200, or 400 cells per well. After 7–10 days, the clonal growth was determined; a colony of>50 cells was considered as one clone. Cells were fixed using 4% paraformaldehyde and stained using crystal violet (Solarbio, Beijing, China). The clone formation rate was calculated as follows: clone formation rate (%) = (clone number/incubation cell number) × 100%.

### Wound-healing assay

Cells were seeded into six-well plates 24 h before the assay, and cultured to 70% confluency according to Lipofectamine 3000 instructions. Wounds of a 2 mm width were created using a plastic scriber. After 24 h and 48 h, the cultures were observed using a microscope (Olympus, Tokyo, Japan).

### Migration and invasion assay

Cells were placed in transwell inserts (Millipore, Billerica, Massachusetts, USA) in serum-free RPMI 1640 medium with (for invasion) or without (for migration) 30 μL of matrigel-coated membrane (BD Biosciences, San Jose, California, USA). A total of 900 μL of medium containing 10% FBS was added to the lower compartment. After incubation for 48 h at 37 °C, cells were fixed using 4% paraformaldehyde and stained using crystal violet.

### Flow cytometric analysis of cell cycle and apoptosis

For cell cycle analysis, 1 mL of DNA staining solution (Multi Sciences, Hangzhou, China) and 10 μL of permeabilization solution (Multi Sciences, Hangzhou, China) were added. The cultures were analyzed using a COULTER-XL.MCL system (Beckman Coulter, Brea, California, USA) after incubation for 30 min. For apoptosis analysis, cells were resuspended in moderate binding buffer containing Annexin V–fluorescein isothiocyanate (FITC) with propidium iodide (PI) (Biolegend, San Diego, California, USA) or Annexin V–phycoerytherin(V-PE) with 7-aminoactinomycin D (7-AAD) (Multi Sciences, Hangzhou, China). The samples were analyzed after incubation for 15 min and results were obtained using MultiCycle and EXP032.

### Label-free time-lapse holographic imaging and analysis

Cell morphology was studied using a label-free technique and digital holographic microscopy using a HoloMonitor M4 (Phase Holographic Imaging, Lund, Sweden). Cells were tracked for 96 h by imaging at 15 min intervals. All indices were analyzed using HoloMonitor (HoloStudio).

### Immunofluorescence

Cells were fixed using 4% formaldehyde and permeabilized using 0.5% Triton X-100 (BioFroxx, Kronbergim Taunus, Germany). Samples were blocked using 5% bovine serum albumin (ComWin Biotech, Beijing, China) and incubated with primary antibody at 4 °C overnight, and a secondary antibody, DyLight 594 (Abbkine, Wuhan, China), for 1 h. Nuclei were counterstained using 4′,6-diamidino-2-phenylindole (DAPI) (Solarbio, Beijing, China) for 10 min. Images were captured during confocal microscopy.

### Immunohistochemical staining

Blocks were sectioned into slices and placed into dimethylbenzene for 15 min, absolute ethyl alcohol for 15 min, absolute ethyl alcohol for another 5 min, 85% ethyl alcohol for 5 min, and 75% ethyl alcohol for 5 min. The samples were then treated using 3% H_2_O_2_ in methanol, followed by incubation with 5% bovine serum albumin for 30 min, a BCAP31 primary antibody at 4 °C overnight, and a horseradish peroxidase-conjugated secondary antibody (ComWin Biotech, Beijing, China) for 50 min. The samples were visualized using 3, 3′-diaminobenzidine and hematoxylin stains.

### Evaluation of staining

Immunohistochemical staining was scored according to intensity, as follows: ‒ (negative), 1+ (weak), 2+ (moderate) or 3+ (strong). Samples were graded based on the staining intensity of the overall proportion of cell staining intensity^49^: −, no expression, no stained cells observed; 1+, weak expression, 1–33% of cells stained; 2+, moderate expression, 34–66% of cells stained; and 3+, strong expression, 67–100% of cells stained. Expression was considered positive when the score was ≥2.

### Mass spectrometry

A total of 10 tissues, as well as A549 and PLA-801D cells, and their respectively stably transfected cell lines, were treated using RIPA lysis buffer, and mass spectrometric analysis was performed by Key Laboratory of Oncology, the Fourth Military Medical University.

### Statistical analysis

Correlations between categorical and continuous variables were assessed using the χ^2^, Fisher’s exact test, and independent *t* test, respectively. Median follow-up times were estimated among surviving patients. All survival times were analyzed using the Kaplan–Meier method. The log-rank test was used to study the correlation of potential prognostic variables with survival times. Data are presented as mean values ± standard error of the mean (SEM). Differences were considered as being significant when *p* < 0.05. All statistical analyses were performed using SPSS 22.0 software.

### Ethics approval and consent to participate

The present study was approved by the Ethics Committee of the Fourth Military Medical University.

### Consent for publication

Consent for publication has been obtained from the patients.

## Supplementary information

Supplementary Information.

Supplementary Table S2.

Supplementary Video S1.

Supplementary Video S2.

Supplementary Video S3.

Supplementary Video S4.

Supplementary Video S5.

Supplementary Video S6.

Supplementary Video S7.

Supplementary Video S8.

Supplementary Video S9.

Supplementary Video S10.

Supplementary Video S11.

Supplementary Video S12.

Supplementary Video S13.

Supplementary Video S14.

Supplementary Video S15.

Supplementary Video S16.

Supplementary Video S17.

Supplementary Video S18.

Supplementary Video S19.

Supplementary Video S20.

Supplementary Video S21.

Supplementary Video S22.

Supplementary Video S23.

## References

[1] Ferlay J (2015). Cancer incidence and mortality worldwide: Sources, methods and major patterns in GLOBOCAN 2012. Int. J. Cancer..

[2] He Y (2018). XRCC3 Thr241Met and TYMS variable number tandem repeat polymorphisms are associated with time-to-metastasis in colorectal cancer. PLoS One..

[3] Kulkarni P, Uversky VN (2017). Cancer/testis antigens: “smart” biomarkers for diagnosis and prognosis of prostate and other cancers. Int. J. Mol. Sci..

[4] Gaugler B (1994). Human gene MAGE-3 codes for an antigen recognized on a melanoma by autologous cytolytic T lymphocytes. J. Exp. Med..

[5] John T (2013). The role of Cancer-Testis antigens as predictive and prognostic markers in non-small cell lung cancer. PLoS One..

[6] Tajima K (2003). Expression of cancer/testis (CT) antigens in lung cancer. Lung Cancer..

[7] Scanlan MJ (2000). Expression of cancer-testis antigens in lung cancer: de®nition of bromodomain testis-speci®c gene (BRDT) as a new CT gene, CT9. Cancer Lett..

[8] Shigematsu Y (2010). Clinical significance of cancer/testis antigens expression in patients with non-small cell lung cancer. Lung Cancer..

[9] Maine EA (2016). The cancer-testis antigens SPANX-A:C:D and CTAG2 promote breast cancer invasion. Oncotarget..

[10] Mirandola L (2015). Novel antigens in non-small cell lung cancer: SP17, AKAP4, and PTTG1 are potential immunotherapeutic targets. Oncotarget..

[11] Dang E (2018). BAP31, a newly defined cancer/testis antigen, regulates proliferation, migration, and invasion to promote cervical cancer progression. Cell Death Dis..

[12] Ng FW (1997). p28 Bap31, a Bcl-2:Bcl-XL- and procaspase-8–associated protein in the endoplasmic reticulum. J. Cell Biol..

[13] Bartee E (2010). Membrane-Associated RING-CH proteins associate with Bap31 and target CD81 and CD44 to lysosomes. PLoS One..

[14] Albanyan S, Al Teneiji A, Monfared N, Mercimek-Mahmutoglu S (2017). BCAP31-associated encephalopathy and complex movement disorder mimicking mitochondrial encephalopathy. Am. J. Med. Genet. A..

[15] Elsemman IE, Mardinoglu A, Shoaie S, Soliman TH, Nielsen J (2016). Systems biology analysis of hepatitis C virus infection reveals the role of copy number increases in regions of chromosome 1q in hepatocellular carcinoma metabolism. Mol. Biosyst..

[16] Benevolenskaya EV (2016). DNA methylation and hormone receptor status in breast cancer. Clin. Epigenetics..

[17] Ye J (2017). ERp29 controls invasion and metastasis of gastric carcinoma by inhibition of epithelial-mesenchymal transition via PI3K/Aktsignaling pathway. BMC Cancer..

[18] Nami B, Donmez H, Kocak N (2016). Tunicamycin-induced endoplasmic reticulum stress reduces *in vitro* subpopulation and invasion of CD44+/CD24- phenotype breast cancer stem cells. Exp. Toxicol. Pathol..

[19] Zheng HC, Gong BC, Zhao S (2017). The meta and bioinformatics analysis of GRP78 expression in gastric cancer. Oncotarget..

[20] Lee HY (2015). GRP78 Protein Expression as Prognostic Values in Neoadjuvant Chemoradiotherapy and Laparoscopic Surgery for Locally Advanced Rectal Cancer. Cancer Res. Treat..

[21] Kim WT, Seo Choi H, Min Lee H, Jang YJ, Ryu CJ (2014). B-cell receptor-associated protein 31 regulates human embryonic stem cell adhesion, stemness, and survival via control of epithelial cell adhesion molecule. Stem Cells..

[22] Sheng W (2017). Calreticulin promotes EGF-induced EMT in pancreatic cancer cells via Integrin/EGFR-ERK/MAPK signaling pathway. Cell Death Dis..

[23] Dai YJ (2018). Concomitant high expression of ERα36, GRP78 and GRP94 is associated with aggressive papillary thyroid cancer behavior. Cell Oncol..

[24] Jiang L, Deberardinis R, Boothman DA (2015). The cancer cell ‘energy grid’: TGF- β1 signaling coordinates metabolism for migration. Mol. Cell Oncol..

[25] Bitsouni V, Trucu D, Chaplain MAJ, Eftimie R (2018). Aggregation and travelling wave dynamics in a two-population model of cancer cell growth and invasion. Math. Med. Biol..

[26] Qian Y (2004). PI3K induced actin filament remodeling through Akt and p70S6K1: implication of essential role in cell migration. Am. J. Physiol. Cell Physiol..

[27] Zhu Y (2018). Chelerythrine Inhibits Human Hepatocellular Carcinoma Metastasis *in Vitro*. Biol. Pharm. Bull..

[28] Lee H, Kim JS, Kim E (2012). Fucoidan from seaweed Fucus vesiculosus inhibits migration and invasion of human lung cancer cell via PI3K-Akt-mTOR pathways. PLoS One..

[29] Bracho-Valdés I (2011). mTORC1- and mTORC2-interacting proteins keep their multifunctional partners focused. IUBMB Life..

[30] Fischer KR (2015). Epithelial-to-mesenchymal transition is not required for lung metastasis but contributes to chemoresistance. Nature..

[31] Pene F (2002). Role of the phosphatidylinositol 3-kinase:Akt and mTOR:P70S6-kinase pathways in the proliferation and apoptosis in multiple myeloma. Oncogene..

[32] Courtnay R (2015). Cancer metabolism and the Warburg effect: the role of HIF-1 and PI3K. Mol. Biol. Rep..

[33] Moore J, Megaly M, MacNeil AJ, Klentrou P, Tsiani E (2016). Rosemary extract reduces Akt/mTOR/p70S6K activation and inhibits proliferation and survival of A549 human lung cancer cells. Biomed. Pharmacother..

[34] Ranjan A, Iwakuma T (2016). Non-Canonical Cell Death Induced by p53. Int. J. Mol. Sci..

[35] Breckenridge DG, Nguyen M, Kuppig S, Reth M, Shore GC (2002). The procaspase-8 isoform, procaspase-8L, recruited to the BAP31 complex at the endoplasmic reticulum. Proc. Natl Acad. Sci. USA.

[36] Breckenridge DG, Stojanovic M, Marcellus RC, Shore GC (2003). Caspase cleavage product of BAP31 induces mitochondrial fission through endoplasmic reticulum calcium signals, enhancing cytochromec release to the cytosol. J. Cell Biol..

[37] Seo SR (2017). Enhanced expression of cell-surface B-cell receptor-associated protein 31 contributes to poor survival of non-small cell lung carcinoma cells. PLoS One..

[38] Namba T (2013). CDIP1-BAP31 complex transduces apoptotic signals from endoplasmic reticulum to mitochondria under endoplasmic reticulum stress. Cell Rep..

[39] Hanahan D, Weinberg RA (2011). Hallmarks of cancer: the next generation. Cell..

[40] Zhou M, Shen S, Zhao X, Gong X (2017). Luteoloside induces G0/G1 arrest and pro-death autophagy through the ROS-mediated AKT/mTOR/p70S6K signalling pathway in human non-small cell lung cancer cell lines. Biochem. Biophys. Res. Commun..

[41] Yip PY (2015). Phosphatidylinositol 3-kinase-AKT-mammalian target of rapamycin (PI3K-Akt-mTOR) signaling pathway in non-small cell lung cancer. Transl. Lung Cancer Res..

[42] Wang B (2008). BAP31 Interacts with Sec. 61 Translocons and Promotes Retrotranslocation of CFTRΔF508 via the Derlin-1 Complex. Cell..

[43] Schardt JA, Weber D, Eyholzer M, Mueller BU, Pabst T (2009). Activation of the unfolded protein response is associated with favorable prognosis in acute myeloid leukemia. Clin. Cancer Res..

[44] Mondal D, Mathur A, Chandra PK (2016). Tripping on TRIB3 at the junction of health, metabolic dysfunction and cancer. Biochimie..

[45] Maurel M (2015). Controlling the unfolded protein response-mediated life and death decisions in cancer. Semin. Cancer Biol..

[46] Kim SY, Kyaw YY, Cheong J (2017). Functional interaction of endoplasmic reticulum stress and hepatitis B virus in the pathogenesis of liver diseases. World J. Gastroenterol..

[47] Corazzari M, Gagliardi M, Fimia GM, Piacentini M (2017). Endoplasmic Reticulum Stress, Unfolded Protein Response, and Cancer Cell Fate. Front. Oncol..

[48] UyBico SJ (2010). Lung cancer staging essentials: the new TNM staging system and potential imaging pitfalls. Radiographics..

[49] Nieto Y, Nawaz F, Jones RB, Shpall EJ, Nawaz S (2007). Prognostic significance of overexpression and phosphorylation of epidermal growth factor receptor (EGFR) and the presence of truncated EGFRvIII in locoregionally advanced breast cancer. J. Clin. Oncol..

