# Supplementary material for: BCAP31, a cancer/testis antigen-like protein, can act as a probe for non-small-cell lung cancer metastasis
Source: Sci Rep. 2020 Mar 4;10:4025. doi: 10.1038/s41598-020-60905-7 (PMC7055246; doi:10.1038/s41598-020-60905-7)
Supplement: Supplementary file 1 — Supplementary Information. [file 41598_2020_60905_MOESM1_ESM.pdf]

Supplementary Information

BCAP31, a cancer/testis antigen-like protein, can act as a probe for non-small-cell lung cancer metastasis

Author:
Jing Wang[+1], Dongbo Jiang[+1], Zichao Li[+1], Shuya Yang[+1], Jiayi Zhou[1], Guanwen Zhang[1], Zixin Zhang[1], Yuanjie Sun[1], Zhipei Zhang[2], Xiaofei Li[2], Liang Tao[1], Jingqi Shi[1], Yuchen Lu[1], Lianhe Zheng[3], Chaojun Song*[1], Kun Yang*[1]
+Co-first authors. These authors contributed equally to this work.
*Corresponding author

Affiliations:

1 Department of Immunology; 2 Department of Thoracic Surgery, Tangdu Hospital; 3 Department of Orthopedics, Tangdu Hospital; the Fourth Military Medical University, No.169, Changle W. Rd., Xi'an 710032, China

List of supplementary material:

1 Supplementary Figure S1. BCAP31 mRNA expression had no effect on NSCLC prognosis.
2 Supplementary Figure S2. B cell receptor-associated protein 31 (BCAP31) did not significantly affect proliferation, cell cycle and apoptosis in three NSCLC cell types.
3 Supplementary Table S1. Seven transcript expression microarray datasets extracted from the Gene Expression Omnibus (GEO).
4 Supplementary Table S2. GSEA reports of BCAP31-associated molecules, biological processes and pathways (sheets 1–4) and label free MS of analysis for mechanism exploration demonstrated the involvement of BCAP31 in primitive functions (sheets 5–6).
5 Supplementary Video S1- S23. The HoloMonitor M4 recorded cell states by real-time monitoring. The videos from S1-S23 were respectively for A549, AN, AS, AN', AO, ALN, ALS, ALN', ALO, PLA-801D, PN, PS, PN', PO, PLN, PLS, PLN', PLO, GLC-82, GN, GS, GN', GO.

Legends:

Supplementary Figure S1.

BCAP31 mRNA expression had no effect on NSCLC prognosis.

A meta-analysis of the relationship between BCAP31 and patients, and the overall survival curve. Both showed that BCAP31 was not related to NSCLC patient survival at the mRNA level.

Supplementary Figure S2.

BCAP31 did not significantly affect proliferation, cell cycle, and apoptosis in three NSCLC cell types.

(**A**) GSEA database analysis of the BCAP31 relationship with cell cycle and apoptosis.

(**B**) Flow cytometry images of cell cycle at 24 h, 48 h, and 72 h after BCAP31 expression was changed using transient transfection. Data were analyzed using a *t* test in each group, and the results were not statistically significant.

(**C**) Flow cytometry images of cell apoptosis at 24 h, 48 h, and 72 h after BCAP31 expression was knocked down using transient transfection. Data were analyzed using a *t* test in each group, and the results were not statistically significant.

(**D**) After transient transfections, clone formation assays of three NSCLC cell types were performed, using 100, 200, and 400 cells seeded in each well of a 6-well plate. Means were calculated and analyzed using a *t* test in each group, and the results were not statistically significant.

(**E**) Cell proliferation (non-transfected and transiently transfected cells) was recorded using an xCELLigence RTCA DP instrument and showed a similar trend as the clone formation assays.

(**F**) In a HoloMonitor M4 irregularity recording of NSCLC cells, which represents cell activity, each cell strain had an stable level of irregularity showing a good condition.

(**G**) In a HoloMonitor M4 optical thickness recording of NSCLC cells, the results were irregular in different cell lines.

AN, PN, GN: control groups of decreased BCAP31 expression with transient transfection in three cell lines; AS, PS, GS: experimental groups of decreased BCAP31 expression with transient transfection in three cell lines; AN’, PN’, GN’: control groups of increased BCAP31 expression with transient transfection in three cell lines; AO, PO, GO: experimental groups of increased BCAP31 expression with transient transfection in three cell lines. All experiments were repeated at least three times.

Study or Subgroup	High expression of BCAP31 Events	Total	Low expression of BCAP31 Events	Total	Weight	Risk Ratio M-H, Random, 95% CI
GSE11969	33	64	30	65	36.1%	1.12 [0.78, 1.59]
GSE42127	30	88	34	88	29.4%	0.88 [0.60, 1.31]
GSE50081	33	86	37	86	34.5%	0.89 [0.62, 1.28]
**Total (95% CI)**		**238**		**239**	**100.0%**	**0.96 [0.78, 1.19]**
Total events	96		101			

Heterogeneity: Tau² = 0.00; Chi² = 1.05, df = 2 (P = 0.59); I² = 0%

Test for overall effect: Z = 0.34 (P = 0.74)

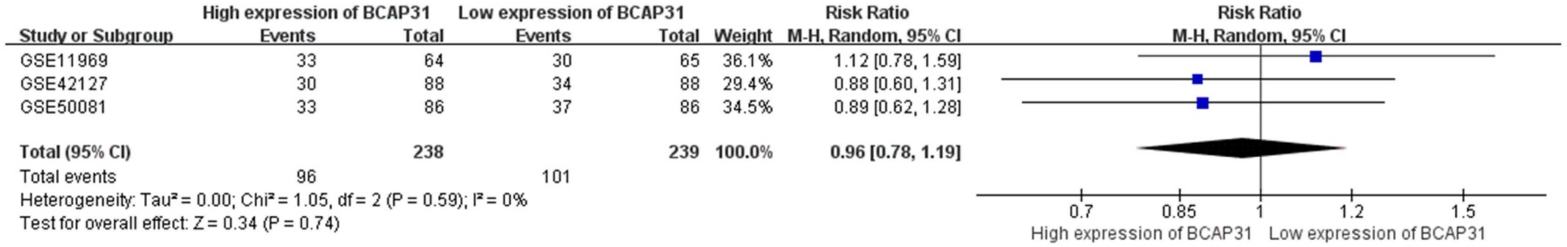

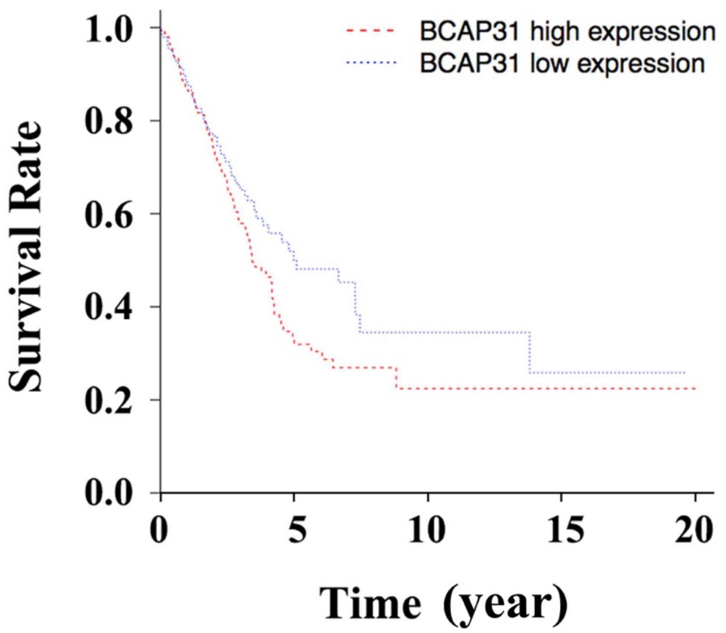

Supplementary Figure S1

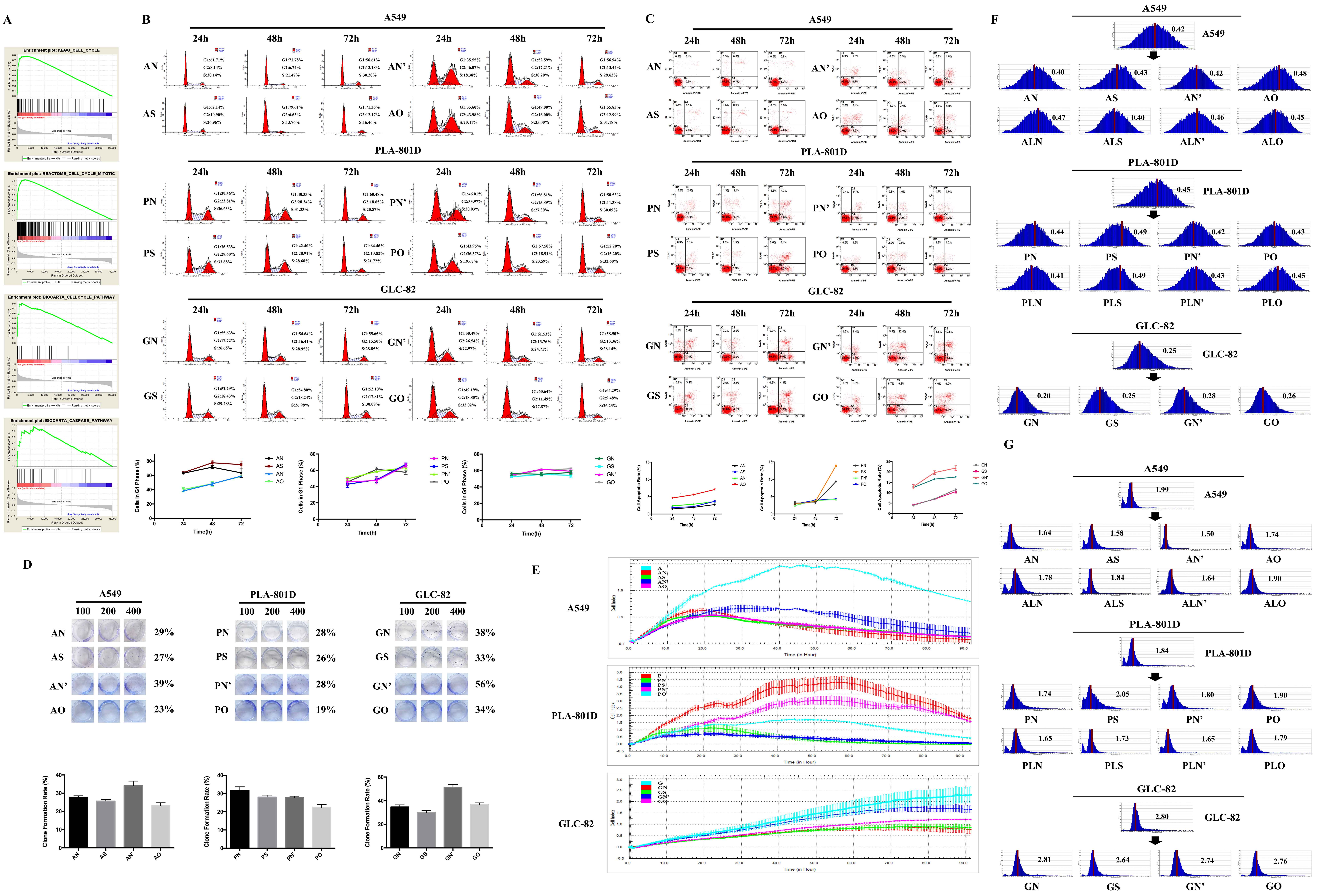

A
B
C
D
E
F
G
A549
PLA-801D
GLC-82
24h
48h
72h
AN
AS
AN'
AO
PN
PS
PN'
PO
GN
GS
GN'
GO
ALN
ALS
ALN'
ALO
PLN
PLS
PLN'
PLO
100
200
400
29%
27%
39%
23%
28%
26%
28%
19%
38%
33%
56%
34%
0.42
0.40
0.43
0.42
0.48
0.47
0.40
0.46
0.45
0.45
0.44
0.49
0.42
0.43
0.41
0.49
0.43
0.45
0.25
0.20
0.25
0.28
0.26
1.99
1.64
1.58
1.50
1.74
1.78
1.84
1.64
1.90
1.84
1.74
2.05
1.80
1.90
1.65
1.73
1.65
1.79
2.80
2.81
2.64
2.74
2.76
Cells in G1 Phase (%)
Time(h)
Cell Apoptotic Rate (%)
Clone Formation Rate (%)
Cell Index
Time (in Hour)

Supplementary Figure S2

Supplementary Table S1. Clinical Relevance of BCAP31 from Gene Expression Omnibus

		GSE11969 (n=129)			GSE13255 （n=137）			GSE14814 (n=133)			GSE33532 （n=80）			GSE42127 (n=176)			GSE50081 （n=172）			GSE103512 (n=55)			Total		
BCAP31		High	Low	P	High	Low	P	High	Low	P	High	Low	P	High	Low	P	High	Low	P	High	Low	P	High	Low	P
Relapse	Y	36	32	.4246													23	26	.6123				59	58	.8696
	N	28	33														63	60					91	93	
Status	Alive	31	35	.5389										58	54	.5308	53	49	.5347				142	139	.6697
	Dead	33	30											30	34		33	37					96	101	
Gender	F	21	24	.6243	31	37	.3470	21	21	.9530	12	4	.0253	43	40	.6505	23	37		5	14	.0141	156	177	.1758
	M	43	41		37	32		45	46		28	36		45	48		63	49		22	14		283	266	
Age	<60	24	26	.7707	17	23	.2834	17	27	.2520	5	11	.0935				17	8	.0515	10	10	.9188	90	105	.3497
	>60	40	39		51	46		39	40		35	29					69	78		17	18		251	250	
Smoking	Y	21	25	.5030	62	66	.2904																83	91	.3883
	N	43	40		6	3																	49	43	
Histology	AD	35	55	.0006							19	21	.6015	62	71	.1144	62	67	.3786	11	19	.0435	189	233	.0009
	SQ	27	8								7	9		26	17		24	19		16	9		100	62	
	Mix	2	2								14	10		0	0		0	0		0	0		16	12	
K-Ras	WT	61	58	.1965																			61	58	.1965
Status	MuT	3	7																				3	7	
P53	WT	30	46	.0058																			30	46	.0058
Status	MuT	34	19																				34	19	
EGFR	WT	50	46	.3384																			50	46	.3384
Status	MuT	14	19																				14	19	
COPD	Y				30	33	.6632																30	33	.6632
	N				38	36																	38	36	

Y: Yes; N: No; F: Female; M: Male; AD: Adenocarcinoma; SQ: Squamous cell carcinoma; WT: Wild type; MuT: Mutant type; EGFR: Epidermal Growth Factor Receptor; COPD: Chronic Obstructive Pulmonary Disease
